# Ototoxicity of boric acid powder in a rat animal model^☆^

**DOI:** 10.1016/j.bjorl.2017.03.010

**Published:** 2017-04-22

**Authors:** Murat Salihoglu, Salim Dogru, Enver Cesmeci, Halil Caliskan, Onuralp Kurt, Zafer Kuçukodaci, Atila Gungor

**Affiliations:** a GATA Haydarpasa Training Hospital, Department of Otorhinolaryngology, Istanbul, Turkey; b Efes Otolaryngology Branch Center, Department of Otorhinolaryngology, Izmir, Turkey; c Diyarbakir Military Hospital, Department of Otorhinolaryngology, Diyarbakir, Turkey; d Eskisehir Military Hospital, Department of Otorhinolaryngology, Eskisehir, Turkey; e Erzincan Military Hospital, Department of Otorhinolaryngology, Erzincan, Turkey; f GATA Haydarpaşa Training Hospital, Department of Pathology, Istanbul, Turkey

**Keywords:** Boric acid, Ear, Otoacoustic emissions, Rats, Ácido bórico, Ouvido, Emissões otoacústicas, Ratos

## Abstract

**Introduction:**

Boric acid, which has antiseptic and acidic properties, is used to treat external and middle ear infections. However, we have not found any literature about the effect of boric acid powder on middle ear mucosa and inner ear.

**Objective:**

The purpose of this study is to investigate possible ototoxic effects of boric acid powder on cochlear outer hair cell function and histological changes in middle ear mucosa in a rat animal model.

**Methods:**

Twenty healthy, mature Wistar albino rats were used in this study. The rats were divided into two groups, Group A and Group B, each of which consisted of 10 rats. Initially, the animals in each group underwent distortion product otoacoustic emissions testing of their right and left ears. After the first distortion product otoacoustic emissions test, a surgical microscope was used to make a small perforation in both ears of the rats in each group, and a second distortion product otoacoustic emissions test was used to measure both ears in all of the rats. Boric acid powder was applied to the right middle ear of the rats using tympanic membrane perforation, and the distortion product otoacoustic emissions were measured immediately after the boric acid powder application. The histological changes and distortion product otoacoustic emissions were evaluated three days later in Group A and 40 days later in Group B.

**Results:**

No significant differences were found at all of the distortion product otoacoustic emissions frequencies. In Group A, mild inflammation of the middle ear mucosa was found on the third day after boric acid powder application. In Group B, boric acid powder caused mild inflammatory changes on the 40th day, which declined over time. Those changes did not lead to significant fibrosis within the mucosa.

**Conclusion:**

In rats, boric acid powder causes mild inflammation in middle ear mucosa and it has no ototoxic effects on cochlear outer hair cell function in the inner ear of rats.

## Resumo

**Introdução:**

O ácido bórico, que tem propriedades antissépticas e ácidas, é usado para tratar infecções de orelha externa e média. No entanto, não encontramos nenhuma literatura sobre o efeito do ácido bórico em pó sobre a mucosa da orelha interna e da mucosa da orelha média.

**Objetivo:**

Investigar possíveis efeitos ototóxicos do ácido bórico em pó sobre a função das células ciliadas externas cocleares e alterações histológicas na mucosa da orelha média em um modelo animal de rato.

**Método:**

Vinte ratos Wistar albinos maduros e saudáveis foram utilizados neste estudo. Os ratos foram divididos em dois grupos, Grupo A e Grupo B, cada um dos quais com 10 ratos. Inicialmente, os animais de cada grupo foram submetidos a testes de emissões otoacústicas–produto de distorção, nas orelhas direita e esquerda. Após o primeiro teste de emissões otoacústicas - produto de distorção, utilizou-se um microscópio cirúrgico para fazer uma pequena perfuração em ambas as orelhas dos ratos em cada grupo, e um segundo teste de emissões otoacústicas - produto de distorção foi utilizado para medir e avaliar as orelhas em todos os ratos. O ácido bórico em pó foi aplicado orelha média direita dos ratos utilizando perfuração da membrana timpânica e as emissões otoacústicas - produto de distorção foram medidas imediatamente após a aplicação de ácido bórico em pó. As alterações histológicas e emissões otoacústicas - produto de distorção foram avaliadas três dias depois no Grupo A e 40 dias depois no Grupo B.

**Resultados:**

Não foram encontradas diferenças significativas em todas as frequências da emissões otoacústicas - produto de distorção. No Grupo A, foi observada uma ligeira inflamação da mucosa da orelha média no terceiro dia após a aplicação de ácido bórico em pó. No Grupo B, o ácido bórico em pó causou leves alterações inflamatórias após 40 dias, que diminuíram ao longo do tempo. Essas alterações não levaram à fibrose significativa da mucosa.

**Conclusão:**

Em ratos, o ácido bórico em pó causa inflamação leve na mucosa da orelha média e não tem efeitos ototóxicos na função das células ciliadas externas da cóclea na orelha interna.

## Introduction

In addition to commercially available drugs containing antibiotics and steroids, many topical agents are used to treat ear diseases. Antiseptic and acidic ear drops are often used in cases of middle ear inflammation and infection, which accompany external ear infections and tympanic membrane perforations.^1^ Topical applications have some advantages in otology. They provide a high concentration of medication in the applied regions, they have less potential to develop bacterial resistance, and they do not cause systemic side effects in patients. In spite of these advantages, after application to the middle ear cavity they can pass through the round window and they could have adverse effects on the cochlear and vestibular apparatus.^2^ Studies have demonstrated the ototoxic effects of aminoglycoside group medications such as gentamycin and streptomycin.^3^ In addition, some antiseptic solutions have been proven to have ototoxic potential.^4^, ^5^ During topical medication, ototoxicity can be caused either by the active substance itself or by the carrier solution. Furthermore, the concentrations of the active substance and the carrier solution affect the ototoxicity.^5^

Boric acid (also known as Boracic) is a white crystalline solid with the molecular formula H_3_BO_3_. It is a weak acid found in nature (minerals, sea water, and fruits), and it can be produced by reacting borate minerals with sulfuric acid. Boracic has various uses as an insecticide, preservative, lubricant, and industrial agent. Medicinally, it is used as an antiseptic; for example, it is diluted as an eye wash, or it used to treat minor cuts and burns, acne, aphthous lesions, and ulcerated diphtheria lesions, and it is used to treat cases involving flux, such as gonorrhea vaginitis, and cystitis. It is also used to treat fungal and bacterial infections in the external or middle ear. It has been known to be toxic in high doses, especially in infants. It is frequently used as a 4% solution prepared with 70% alcohol or distilled water, and in pure powder form it is used as Boric Acid Powder (BAP).^6^, ^7^ BAP contains the highest concentration of boric acid that is used in ototopical medication. To date, the effects of BAP on the inner and middle ear have not been evaluated. This present study investigated the possible ototoxic effects of BAP on the cochlear outer hair cell function in rats by measuring Distortion Product Otoacoustic Emission (DPOAE) amplitudes. Histological changes within the middle ear mucosa were also evaluated.

## Methods

Twenty healthy, mature (16–20 month-old), Wistar albino rats (weighing between 250 g and 300 g) were used in this study. The experimental protocol was designed according to the Guide for the Care and Use of Laboratory Animals published by the National Academics Press.^8^ The rats were kept in separate cages in a room with 12 h on/off light cycles, simulating the standard day and night rhythm, at constant temperature (20 ± 2 °C) and humidity (55.5%). This study was approved by the Ethics Committee on the University Hospital (protocol number 58-2013). The rats were anesthetized with ketamine (75–90 mg/kg, Ketalar, Pfizer, Istanbul, Turkey) and xylazine (5–8 mg/kg, Rompun, Bayer, Leverkusen, Germany) through intraperitoneal injection. The depth of anesthesia was determined by pedal reflex, and additional anesthesia was administered in half dose increments as required. The rats were euthanized with thiopental sodium (200 mg/kg Pentothal; Abboth, Campoverde di Aprilla, Italy) through intraperitoneal injection under anesthesia.

The rats were divided into two groups, Group A and Group B, each consisting of 10 animals (five males, five females). Following the administration of general anesthesia, we assessed the external ear canal and the tympanic membrane of each rat using a surgical microscope. None of the rats had an ear infection or an ear injury. Initially, the animals in each group underwent DPOAE testing in the right and left ears. After this first DPOAE testing, a surgical microscope was used to make a small (less than one-quarter of the tympanic membrane) perforation in both ears of the rats in each group, and a second DPOAE test was used to measure both ears for all of the rats in each group. Then, 35 mg (0.1 cc) BAP was applied to the right middle ear of the rats in both groups via tympanic membrane perforation using a 2.5 cc syringe with a 22 gauge needle. After this procedure, the middle ear mucosa was observed through the perforation using microscope in order to ensure that BAP in the middle ear mucosa. DPOAE testing was repeated on the right ears of the rats in both groups immediately after the BAP application. Histological changes and DPOAEs were evaluated in both ears three days later in Group A and forty days later in Group B. The histological results and the DPOAE measurements were compared between the right and left ears, and between both groups.

DPOAE thresholds were recorded at 2 kHz, 3 kHz, 4 kHz, 6 kHz, and 8 kHz using a Madsen Capella cochlear emission analyzer, Noah 3 system (GN Otometrics A/S, Taastrup, Denmark). All measurements were carried out in a quiet room. Primary tones were emitted into the sealed external ear canals through an earphone. The acoustic stimulus that created the DPOAE signal consisted of two simultaneous, continuous pure tones at different frequencies: f1 and f2. In this study, we used the stimulus parameters of 80 dB SPL/70 dB SPL with the f2/f1 ratio of 1.22, and then the amplitude of the DPOAE signal was analyzed. A total of 1000 acquisitions were analyzed.

All of the rats in both groups were euthanized under anesthesia for histologic evaluation. Subsequently, the temporal bones were harvested, and the tympanic bullas were quickly dissected from the temporal bones and prepared for microscopic examination. After fixing in 10% buffered formaline solution for 48 h, the specimens were placed in a 10% nitric acid decalcifying solution for one day. Each specimen was then embedded in paraffin and cut according to the standard histologic technique (Leica microtome, Germany), and then stained with trichrome, hematoxylin, and eosin. The tissues were viewed with a microscope, and photomicrographs were obtained. The histologic changes were evaluated by observing the middle ear mucosa to determine the accumulation of inflammatory cells (polymorphonuclear leukocytes, macrophages, lymphocytes, giant cells, and other cells) and the presence of fibroblastic activity and fibrosis. The degree of these changes was visually assessed and graded as follows: absent (0), mild (1), moderate (2), or severe (3).

The classification of inflammation according to inflammatory cell counts was performed at 200× magnification and the counts were categorized as follows: mild (<50 cells/field), moderate (50–100 cells/field), and severe (>100 cells/field). Ten 200× magnification areas were examined, and the average number of inflammatory cells was calculated.^9^ Fibroblastic activity and fibrosis were graded according to the classification used by Dogru at al.^10^ The histologic observations were performed blinded by the same pathologist who viewed each slide twice, and five measurements were performed for each specimen.

### Statistical analysis

Data analyses were performed using SPSS 21.0 (Statistical Package for Social Sciences, SPSS Inc., Chicago, IL). The data were shown as the mean ± standard deviation for the continuous variables (DPOAE), and as the number of cases for the categorical variables (histologic observations). When determining whether or not the data are normally distributed, we have found non-normal distribution. The Mann–Whitney *U* test was used to compare between the right and left ears in all of the measured DPOAE frequencies in both groups. Wilcoxon rank-sum test was used to compare the DPOAE results for the right ears (the BAP-applied ears) of the rats in Group B, measured three different times (first day; before perforation, after perforation, after boric acid powder application, 3rd day and 40th day) throughout the study. Categorical variables (histologic observations) were compared by Fisher's exact test. Statistical significance was reserved for values (*p* < 0.05).

## Results

None of the rats died during the study. The tympanic membranes of the rats were examined using otomicroscopy before euthanization. In Group A, iatrogenic perforations persisted in both ears of all of the rats, and there were no signs of infection. In Group B, the iatrogenic perforations in both ears were healed in of all of the rats. No significant differences were found between the right and left ears in all of the measured DPOAE frequencies in both groups (Table 1). When we compared the DPOAE results for the right ears (the BAP-applied ears) of the rats in Group B, measured three different times (first day; before perforation, after perforation, after boric acid powder application, 3rd day and 40th day) throughout the study, no statistically significant differences were found (Table 2).

**Table 1:** Distribution of DPOAE amplitudes for 2-, 3-, 4-, 6-, and 8 kHz frequencies in the right and left ears of the rats in Group A and B (80 Db SPL/70 dB SPL stimuli).

	DPOAE, mean ± SD (Group A/B)		
	Right ear	Left ear	*p*-Value^a^
*2* *kHz*			
Before perforation	12.08 ± 0.98/12.35 ± 1.16	12.18 ± 1.10/12.38 ± 0.90	0.70/0.91
After perforation	11.79 ± 0.88/11.82 ± 1.94	11.74 ± 1.19/11.79 ± 1.53	0.76/0.81
After boric acid powder	11.49 ± 1.12/11.21 ± 1.70	–/–	–/–
3rd day	12.06 ± 1.39/12.28 ± 0.88	11.93 ± 1.16/12.34 ± 0.66	0.47/0.80
40th day	–/12.48 ± 1.02	–/12.17 ± 1.10	–/0.76

*3* *kHz*			
Before perforation	23.75 ± 1.87/23.72 ± 1.32	23.23 ± 1.40/23.62 ± 1.72	0.57/0.88
After perforation	23.48 ± 1.73/22.55 ± 1.04	23.07 ± 1.28/22.97 ± 1.24	0.71/0.54
After boric acid powder	23.27 ± 2.11/23.09 ± 1.50	–/–	–/–
3rd day	23.42 ± 1.69/23.72 ± 1.32	23.18 ± 1.77/22.94 ± 1.04	0.82/0.15
40th day	–/23.27 ± 1.18	–/22.99 ± 1.84	–/0.65

*4* *kHz*			
Before perforation	26.28 ± 1.51/26.18 ± 1.88	26.17 ± 2.05/26.08 ± 2.14	0.94/0.53
After perforation	26.22 ± 1.86/26.02 ± 1.60	25.17 ± 1.45/25.62 ± 1.96	0.23/0.47
After boric acid powder	25.97 ± 1.74/25.94 ± 1.95	–/–	–/–
3rd day	25.92 ± 2.09/25.94 ± 1.95	25.84 ± 2.16/26.12 ± 1.77	0.88/0.86
40th day	–/26.16 ± 1.74	–/26.06 ± 1.82	–/0.72

*6* *kHz*			
Before perforation	32.87 ± 2.27/33.76 ± 2.21	32.70 ± 1.75/33.50 ± 1.93	0.79/0.92
After perforation	32.73 ± 2.29/32.88 ± 2.59	32.61 ± 1.90/33.14 ± 2.14	0.91/0.80
After boric acid powder	32.41 ± 1.96/32.68 ± 2.06	–/–	–/–
3rd day	32.94 ± 2.92/33.68 ± 2.18	32.67 ± 2.11/33.12 ± 2.22	0.91/0.54
40th day	–/33.40 ± 1.83	–/33.47 ± 2.80	–/0.84

*8* *kHz*			
Before perforation	37.96 ± 3.37/38.23 ± 2.81	38.09 ± 3.13/38.53 ± 30.0	1.00/0.80
After perforation	37.54 ± 2.14/37.76 ± 2.69	38.1 ± 3.24/38.32 ± 2.94	0.55/0.51
After boric acid powder	37.44 ± 2.63/37.25 ± 4.25	–/–	–/–
3rd day	37.52 ± 2.62/37.24 ± 2.62	37.96 ± 2.58/37.46 ± 2.40	1.00/0.76
40th day	–/37.68 ± 2.75	–/37.59 ± 2.62	–/0.96

DPOAE, distortion product otoacoustic emission; SD, standard deviation; SPL, sound pressure level.

a Mann–Whitney *U* test (*p* < 0.05 was considered significant).

**Table 2:** The comparison (*p*-values^a^) of DPOAE amplitudes for 2-, 3-, 4-, 6-, and 8 kHz frequencies of the right ears of the rats in Group B.

Comparison	2 kHz	3 kHz	4 kHz	6 kHz	8 kHz
I–II	0.73	0.07	0.76	0.36	0.72
I–III	0.09	0.37	0.54	0.28	0.51
I–IV	0.92	1.00	0.51	0.54	0.58
I–V	0.54	0.51	1.00	0.61	0.68
II–III	0.44	0.44	0.96	0.84	0.88
II–IV	0.51	0.07	0.57	0.44	0.59
II–V	0.41	0.20	0.88	0.51	0.88
III–IV	0.22	0.37	0.72	0.24	0.88
III–V	0.10	0.92	0.96	0.28	0.88
IV–V	0.41	0.51	0.48	0.44	0.80

I, first day, before perforation; II, first day, after perforation; III, first day, after boric acid powder application; IV, in follow-up on 3rd day; V, in follow-up on 40th day.

a Wilcoxon rank-sum test (*p* < 0.05 was considered significant).

**Figure 1:**
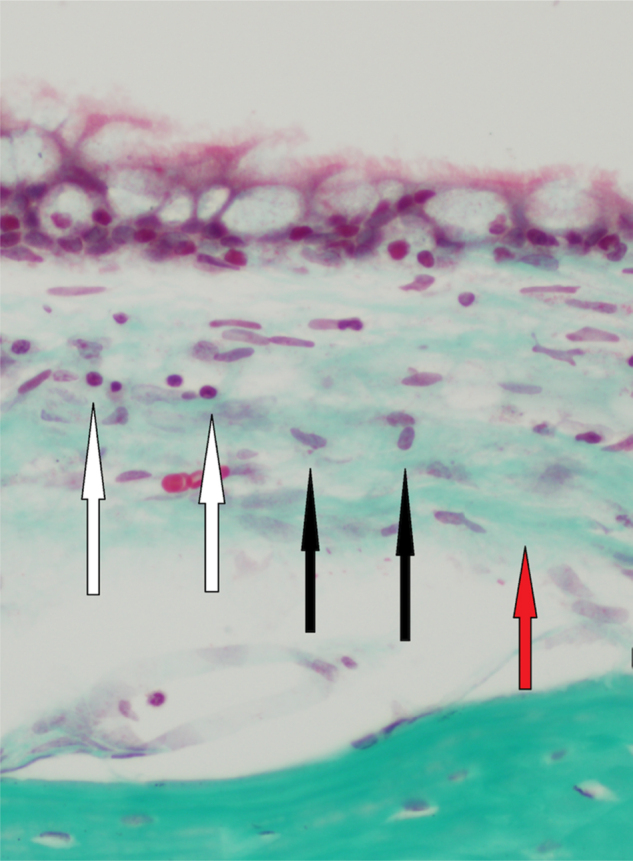
Mild inflammation (white arrows), mild fibroblastic activity (black arrows), and mild fibrosis (red arrow) in one of the rats in Group B (40 days) (Trichrome 400×).

Histopathologic findings in Group A and Group B were as follows (Fig. 1). In Group A, a mild inflammatory cell increase was observed in both ears in eight rats and in the right ear in two rats (*p* > 0.05). In Group B, there was no inflammatory cell increase in eight rats, but there was a mild inflammatory cell increase in the right ear in two rats (*p* > 0.05). In Group A, mild fibroblastic activity was observed in the right ear in three rats (*p* > 0.05). In Group B, mild fibroblastic activity was observed in the right ear in only one rat (*p* > 0.05). No fibrosis was observed in Group A. Mild fibrosis was observed in the right ear in three rats in Group B (*p* > 0.05). The histologic changes of the groups are summarized in Table 3. There was no statistical significant difference in the histologic findings between the right and left middle ear mucosa of the rats in both groups.

**Table 3:** Histologic changes after BAP treatment in the right and left ears in both groups.

Right/left	Absent	Mild	Moderate	Severe	*p*-Value^c^
Inflammatory cells^a^	–/2	10/8	–/–	–/–	0.47
Fibroblastic activity^a^	7/10	3/–	–/–	–/–	0.21
Fibrosis^a^	–/–	–/–	–/–	–/–	–
Inflammatory cells^b^	8/10	2/–	–/–	–/–	0.14
Fibroblastic activity^b^	9/10	1/–	–/–	–/–	0.99
Fibrosis^b^	7/10	3/–	–/–	–/–	0.21

a 3rd day (Group A).

b 40th day (Group B).

c Fisher's exact test (*p* < 0.05 was considered significant).

## Discussion

Topical agents can be used alone or with systemic medications to treat chronic middle ear and external ear canal infections.^11^, ^12^ Many studies have investigated the effects of boric acid and other ototopical solutions that have similar characteristics. Ozcan et al.^13^ reported that a 4% boric acid solution prepared with 70% alcohol is an effective and inexpensive treatment option for otomycosis in addition to local toiled of ear. In a study conducted by Minja et al.^14^ a boric acid solution prepared with 70% alcohol was reported to be effective and safe in children diagnosed with suppurative otitis media. Ozturkcan et al.^6^ investigated the effect of boric acid solutions on Auditory Brainstem Responses (ABR) in guinea pigs. They reported that a 4% boric acid solution prepared with 70% alcohol has an ototoxic effect, whereas the solution is safer if it is prepared with distilled water. Aktas et al.^15^ investigated the ototoxic effect of boric acid solution prepared with different concentrations of alcohol (60% and 40%) by performing ABR on young albino guinea pigs. They reported that a 4% boric acid solution prepared with 60% alcohol had a negative effect on hearing in guinea pigs. They also found that an increase in the concentration of the boric acid solution caused additional ototoxicity. However, Ozdemir et al.^16^ demonstrated that the topical application of boric acid in alcohol solutions to the middle ear of rats was safe for the inner ear. In their study, hearing was evaluated using DPOAE. In addition to boric acid solutions, Burow's solution, which consists of different concentrations of aluminum subacetate, is frequently used as an antiseptic. Serin et al.^17^ effect studied the effects of 4% and 13% Burow's solutions on guinea pig hearing using ABR, and they found no ototoxic effect Gultekin at al.^1^ investigated the effects of Castellani solution, which has antibacterial and antifungal properties, and they did not find any ototoxic effect in rat inner ears using DPOAE.

Previous studies have evaluated the ototoxicity of a 4% (4 g/100 mL) boric acid solution prepared with distilled water or alcohol.^6^, ^13^, ^14^, ^15^, ^16^ However, we have not found any previous studies that have examined the effect of BAP on middle ear mucosa and the inner ear. Another common use of BAP is to insufflate it into the wet tympanic cavities and the external ear. We used 35 mg pure form of BAP (100% concentration) while other studies, mentioned above, used 4% boric acid solution.

Ototoxicity refers to the injury that occurs in the inner ear due to the administration of medications or chemicals resulting in sensorineural hearing loss.^1^ DPOAE studies have been frequently used to investigate ototoxicity. DPOAE is a noninvasive method for evaluating the outer hair cells, which produce Otoacoustic Emissions (OAEs).^18^ However, OAEs can be affected by fluid in the middle ear cavity or the presence of a perforation in the tympanic membrane.^19^ When more than half of the tympanic membrane is perforated, OAEs cannot be measured due to conductive hearing loss.^20^ Therefore, in this present study, the tympanic membranes were partially perforated (less than one-quarter of the tympanic membrane) with the help of a pick, and BAP was applied using a 2.5 cc syringe with a 22 gauge needle.

In our study, no significant differences were observed between the DPOAE levels of the BAP-applied and the non-BAP-applied ears at all frequencies in both groups.

Mild inflammation and fibroblastic activity in the middle ear mucosa were observed three days after the BAP application in Group A. We thought mild inflammation, detected in Group A, was due to traumatic effects of iatrogenic perforation. No fibrosis was seen on the third day after treatment in Group A. Histologic examination of the middle ear mucosa on day 40 revealed that BAP caused mild inflammation, which declined over time in Group B. Those changes led to mild fibrosis in the mucosa in the right ears of only three rats in Group B.

## Conclusions

In our study, we have demonstrated that BAP caused mild histologic changes in middle ear mucosa in rats. We utilized DPOAE to investigate ototoxic effects of BAP to the rat inner ear and found that BAP has no ototoxic effects on cochlear outer hair cell function in the inner ear of rats. It is reasonable to utilize DPOAE to investigate the ototoxicity of drugs.

## Conflicts of interest

The authors declare no conflicts of interest.
